# High Levels of Tumor Necrosis Factor-Alpha Reduce Placental Aquaporin 3 Expression and Impair *in vitro* Trophoblastic Cell Migration

**DOI:** 10.3389/fphys.2021.696495

**Published:** 2021-06-29

**Authors:** Rinaldo Rodrigues dos Passos Junior, Raiany Alves de Freitas, Julieta Reppetti, Yollyseth Medina, Vanessa Dela Justina, Camila Werle Bach, Gisele Facholi Bomfim, Victor Vitorino Lima, Alicia E. Damiano, Fernanda R. Giachini

**Affiliations:** ^1^Institute of Biological Sciences, Federal University of Goias, Goiânia, Brazil; ^2^Faculty of Medicine, Institute of Physiology and Biophysics Bernardo Houssay (IFIBIO)–CONICET, University of Buenos Aires, Buenos Aires, Argentina; ^3^Institute of Biological Sciences and Health, Federal University of Mato Grosso, Barra do Garças, Brazil; ^4^Institute of Health Sciences, Federal University of Mato Grosso, Sinop, Brazil; ^5^Department of Biological Sciences, Faculty of Pharmacy and Biochemistry, University of Buenos Aires, Buenos Aires, Argentina

**Keywords:** placenta, hypertensive pregnancy, preeclampsia, trophoblastic cell, migration

## Abstract

Placentas from preeclamptic women display augmented tumor necrosis factor-alpha (TNF-α) levels with reduced expression of aquaporin 3 (AQP3). However, whether TNF-α modulates AQP3 expression remains to be elucidated. We hypothesize that elevated levels of TNF-α reduce AQP3 expression and negatively impact trophoblastic cell migration. Spontaneously hypertensive rats (SHRs) and Wistar rats (14–16 weeks) were divided into hypertensive and normotensive groups, respectively. Systolic blood pressure (SBP) was measured, and animals mated. In a third group, pregnant SHRs were treated with a TNF-α antagonist, etanercept (0.8 mg/kg, subcutaneously) on days 0, 6, 12, and 18 of pregnancy. Placentas were collected on the 20th day of pregnancy. Human placental explants, from normotensive pregnancies, were incubated with TNF-α (5, 10, and 20 ng/ml) and/or etanercept (1 μg/ml). Swan 71 cells were incubated with TNF-α (10 ng/ml) and/or etanercept (1 μg/ml) and subjected to the wound healing assay. AQP3 expression was assessed by Western blot and TNF-α levels by ELISA. SBP (mmHg) was elevated in the hypertensive group, and etanercept treatment reduced this parameter. Placental TNF-α levels (pg/ml) were higher in the hypertensive group. AQP3 expression was reduced in the hypertensive group, and etanercept treatment reversed this parameter. Explants submitted to TNF-α exposition displayed reduced expression of AQP3, and etanercept incubation reversed it. Trophoblastic cells incubated with TNF-α showed decreased cell migration and reduced AQP3 expression, and etanercept incubation ameliorated it. Altogether, these data demonstrate that high TNF-α levels negatively modulate AQP3 in placental tissue, impairing cell migration, and its relationship in a pregnancy affected by hypertension.

## Introduction

Hypertensive pregnancy is a term commonly used to describe a broad spectrum of conditions, where patients present mild or severe elevations in blood pressure, along with multiple organ dysfunctions (2019). Hypertensive disorders represent the most common complications during pregnancy, ranging around 5–10% of incidence, and are the primary cause of maternal–perinatal mortality and morbidity worldwide (Tooher et al., 2017; Wilkerson and Ogunbodede, 2019). Regarding the hypertensive disorders affecting pregnancies, the most frequent are chronic hypertension, gestational hypertension, preeclampsia (PE) or eclampsia, and chronic hypertension with superimposed PE (Sutton et al., 2018).

The most cited hypothesis about the pathogenesis of hypertensive pregnancies involves reduced placental perfusion in consequence of the inadequate trophoblastic invasion of the myometrium, resulting in poor placental blood supply (hypoperfusion) and diffuse maternal endothelial dysfunction (Braunthal and Brateanu, 2019). However, it is now accepted that a failure in the trophoblast differentiation may lead to hypertensive disorders (Barrientos et al., 2017). Although its etiology remains unclear (Vest and Cho, 2014), there is a consensus that defects in placentation are among the main predisposing factors for PE (Huppertz, 2008; Hawfield and Freedman, 2009).

During placentation, trophoblast cells can differentiate into two different lineages: the villous trophoblast (VT) and the extravillous trophoblast (EVT). While the VT cells cover the chorionic villus and form a syncytium, acting in the fetal–maternal transport, the EVT cells differentiate into interstitial trophoblasts, which can migrate and invade the decidua and the myometrium to transform the maternal spiral arteries. As a result, the spiral arteries acquire the physiological properties necessary to establish adequate maternal blood flow to the developing uteroplacental unit (Davies et al., 2016; Silva and Serakides, 2016).

Tumor necrosis factor-alpha (TNF-α), a pleiotropic cytokine that plays a central role in regulating inflammation, is involved in the pathogenesis of PE, where TNF-α levels are found to be raised in both serum and placental tissue (Weel et al., 2016). Indeed, augmented TNF-α levels during pregnancy are associated with an increased risk for PE development (Zak and Soucek, 2019). Interestingly, TNF-α inhibits trophoblast integration into endothelial cellular networks (Xu et al., 2011) and suppresses the invasion of human trophoblast cells (Wen et al., 2018). However, the exact mechanism behind the TNF-α-mediated reduction of trophoblast cell invasion remains poorly understood.

Lately, the importance of aquaporins (AQPs) during placentation and its importance in fetal development have been recognized (Martinez and Damiano, 2017). Classically, AQPs are water channels that allow the rapid movement of water through the membrane to help maintain homeostasis, also involved in the transport of glycerol (Herrera and Garvin, 2011; Yester and Kuhn, 2017; Delgado-Bermudez et al., 2019). Recently, non-canonical functions of AQPs have also been reported, including proliferation, apoptosis, and cell migration. All these functions are related to temporary changes in cell volume (Kitchen et al., 2015). Remarkably, the expression of AQP3 in PE placentas is reduced (Szpilbarg and Damiano, 2017), and the silencing of AQP3 impairs EVT cell migration (Alejandra et al., 2018) and produces a failure in EVT endovascular differentiation (Reppetti et al., 2020). However, the signal triggering AQP3 reduction during PE is currently unknown.

To address this question, we hypothesized that high TNF-α levels impair cell migration by reducing the expression of AQP3 in placental tissue. In the present study, we investigate whether TNF-α modulates AQP3 in the placenta using a hypertensive pregnancy animal model, as well as an *in vitro* model of human placental explants. Furthermore, we investigate the impact of TNF-α on AQP3 expression and cellular migration in immortalized trophoblast cells from the human placenta.

## Methods

### Animals

All procedures were performed following the Guiding Principles in the Care and Use of Animals, adopted by the Brazilian College of Animal Experimentation. The study was approved by the Committee of Ethics in Animal from the Federal University of Mato Grosso (CEUA #23108.038471/2019-14)

Female Wistar rats and spontaneously hypertensive rats (SHRs) (14–16 weeks old, 180–200 g) were used. The rats were maintained in the animal facility room, at 23 ± 2°C, with 12-h light/dark cycles, fed a standard commercial diet, and free water intake. Blood pressure (BP) was measured by tail-cuff plethysmography after 3 days of acclimatization. To mate, females were housed with Wistar males. Vaginal smears were taken daily, and the day on which spermatozoa were found in the vaginal smear was designated gestational day 0.

### Experimental Design

Pregnant SHRs and Wistar rats were separated into hypertensive (*n* = 6) and normotensive group (*n* = 6), respectively. The third group was composed of pregnant SHRs (*n* = 8), treated with 0.8 mg/kg etanercept (Embrel®), on days 0, 6, 12, and 18 of pregnancy (DOP) *via* subcutaneous injection as performed previously (Small et al., 2016). On the 20th gestational day, rats were anesthetized with 3% sodium pentobarbital (50 mg/kg body weight, intraperitoneally). After laparotomy for removal of placentas, rats were killed by pneumothorax. The fetuses were individually weighed and classified according to the mean values of fetal weights of the normotensive group as small for gestational age [(SGA) fetal weight < Wistar mean - SD × 1.7]; appropriate for gestational age [(AGA) fetal weight within Wistar mean ± SD × 1.7]; and large for gestational age [(LGA) fetal weight > Wistar mean +SD × 1.7] (Damasceno et al., 2013). Fetuses were killed by placement in a CO_2_ chamber.

### Tumor Necrosis Factor-Alpha Measurement

Quantitation of TNF-α from placental tissue was performed by ELISA technique using a commercial kit (TNF-α #558535 BD Biosciences, Pharmingen, San Diego, CA) according to the manufacturer's instructions.

### Human Tissue Collection

This study was approved by the Human Ethics Committee from the Faculty of Pharmacy and Biochemistry of the University of Buenos Aires (EXP-FYB #0045449/2017) and the Ethics Committee from the *Hospital Nacional Dr. Prof. Alejandro Posadas*, Buenos Aires, Argentina. The investigation conforms to the principles outlined in the Declaration of Helsinki. The patients signed written consent forms before sample collection, and clinical data were collected.

Normal human term placenta (*n* = 7) were obtained immediately after vaginal or cesarean delivery (clinical data are shown in Table 1), packed in containers containing ice, and transported to the Reproduction Physiology Laboratory at the University of Buenos Aires.

**Table 1:** Clinical characteristics of normal pregnant women.

**Clinical characteristics**	**Mean ± SEM**
Number of pregnant women	7
Parity	
Primiparous	4
Multiparous	3
Maternal age, years	28.6 ± 2
Gestational age, weeks	38.7 ± 0.4
Mean blood pressure, mmHg	
Systolic	114.3 ± 2
Diastolic	70 ± 2.2
Proteinuria	Negative
Body mass index (BMI), kg/m^2^	25.4 ± 1.4
Birth weight, g	3234 ± 147
Fetal sex	
Male	4
Female	3

*Values are mean ± SEM*.

### Explant Culture

Placental cotyledons were gently separated and washed in 0.9% sodium chloride solution to remove blood from the intervillous spaces. Villous tissue was minced with scalpel blades to obtain explants in the proportion of ~0.5 × 0.5 cm. Explants were cultured in Dulbecco modified Eagle medium (DMEM; Life Technologies, Inc.)/F12 containing 10% fetal bovine serum (FBS), 100 IU/ml penicillin, 100 mg/ml streptomycin, and 32 mg/ml gentamicin at 37°C in a humidified gas mixture of 5% CO_2_ and 95% air.

Explants were placed in a 24-well plate in the proportion of five explants per well. These were then incubated with complete culture medium containing TNF-α at concentrations of 5, 10, or 20 ng/ml, etanercept at a concentration of 1 μg/ml or vehicle (culture medium) for 16 h.

### Explant Viability

Explant viability was verified by the 3-(4,5-dimethylthiazol-2-yl)-2,5-diphenyl tetrazolium bromide (MTT) assay. One explant from each well was removed and transferred to new wells and incubated with 0.5 mg/ml MTT solution (Sigma-Aldrich Corp.) at 37°C for 2 h. Each explant was transferred to a new well, and 100 μl of ethanol was added and incubated for 30 min with shaking at room temperature. Absorbance was measured at 595 nm wavelength.

Explants' toxicity was verified by the release of the intracellular enzyme lactate dehydrogenase (LDH) into the incubation medium after 16 h. Aliquots of the culture medium (500 μl) from each explant were collected and centrifuged. The supernatant was taken (50 μl) and transferred to new wells where they were incubated with a lactate dehydrogenase (LDH) assay solution at room temperature for 30 min. A stop solution was added, and absorbance was measured at 590 nm wavelength.

### Cell Culture

Immortalized human trophoblastic cell line Swan 71, described by Straszewski-Chavez (Straszewski-Chavez et al., 2009), obtained from the 7-week placental cytotrophoblast in the first trimester of a healthy pregnancy, was used. Cells were grown in complete culture medium at 37°C for 24 h in a humidified gas mixture of 5% CO_2_ and 95% air. At confluence, cells were arrested in the corresponding medium supplemented with 0.5% FBS and incubated with 10 ng/ml TNF-α. Cell viability was assessed by the MTT assay as described previously (Alejandra et al., 2018).

### Western Blot Analysis

The total cell lysate was obtained by incubating macerated placenta tissue with lysis buffer containing protease inhibitors, and protein concentration was determined using the Bradford Assay Kit (Sigma-Aldrich). Proteins (50–60 μg) were loaded and separated by electrophoresis on a 12.5% polyacrylamide gel and transferred to a nitrocellulose membrane (Sigma-Aldrich). Non-specific binding sites were blocked with 5% skim dry milk in Tris-buffered saline solution with Tween-20 (TBS-T, pH 7.6) for 1 h at room temperature. Membranes were rinsed and incubated with an anti-AQP3 antibody (1:1,000; Alpha Diagnostic Intl. Inc.) followed by incubation with a peroxidase-conjugated secondary antibody (1:10,000; Jackson ImmunoResearch Laboratories, Inc., West Grove, PA).

Protein bands were detected using the ECL Plus Western Blotting Detection System (GE Healthcare) and then quantified using an image analysis software program (Kodak Digital Science 1D Image Analysis Software, Eastman Kodak, Rochester, NY, USA).

### Wound Healing Assay

Swan 71 cells were cultured in a 24-well plate in complete culture medium at 37°C for 24 h in a humidified gas mixture of 5% CO_2_ and 95% air. Once 90% of cell confluence was observed in the plate wells, a wound was made in each cell monolayer, across the center of the wells, using a 200-μl pipette tip. Then, cells were arrested in the corresponding medium supplemented with 0.5% FBS and incubated with 10 ng/ml TNF-α, 1 μg/ml etanercept, or vehicle (culture medium).

Wounded monolayers were examined at 0, 18, and 24 h and photographed using an optical microscope with a camera attached. Wound closure was measured by calculating the area (mm^2^) using an image analysis software and presented by percentage. The scratch distance at 0 h was considered 0% of gap closure. For migration speed, we measured the width of the wound as the mean distance between the edges of the scratch in each photograph, and this change in mean wound width was divided by the time spent on each migration.

### Statistical Analysis

Statistical analysis was performed, and the data were presented as mean ± standard error of the mean (SEM). The significance of the results was analyzed by Student's *t*-test, one-way ANOVA followed by the Tukey posttest or two-way ANOVA followed by the Bonferroni posttest. For the proportion data, the significance was analyzed by Fisher exact test. *p* < 0.05 was considered statistically significant for all experiments.

## Results

### Fetal Parameters in Spontaneously Hypertensive Rats and Wistar Rats

We assessed the fetal parameters from hypertensive and normotensive pregnant rats. Fetuses from SHRs presented decreased fetal weight (3.4 ± 0.04 g vs. 4.9 ± 0.1 g; *p* < 0.0001) and higher proportion of fetuses classified as SGA (100 vs. 1.5%; *p* = 0.000) compared to Wistar rats (Table 2), indicating impairment of fetal development during the hypertensive pregnancy.

**Table 2:** Fetal parameters from Wistar rats and SHRs.

	**Wistar rat**	**SHR**
Fetal weight (g)	4.9 ± 0.1	3.4 ± 0.04^*^
SGA (%)	1.5	100.0^*^
AGA (%)	97.0	0.0^*^
LGA (%)	1.5	0.0

*SHR, spontaneously hypertensive rats; SGA, small for gestational age; AGA, appropriate for gestational age; LGA, large for gestational age*.

* *p < 0.05 vs. Wistar. Student's unpaired t-test and Fisher exact test (%)*.

### Placental Tumor Necrosis Factor-Alpha Levels and Blood Pressure in Spontaneously Hypertensive Rats and Wistar Rats

**Figure 1:**
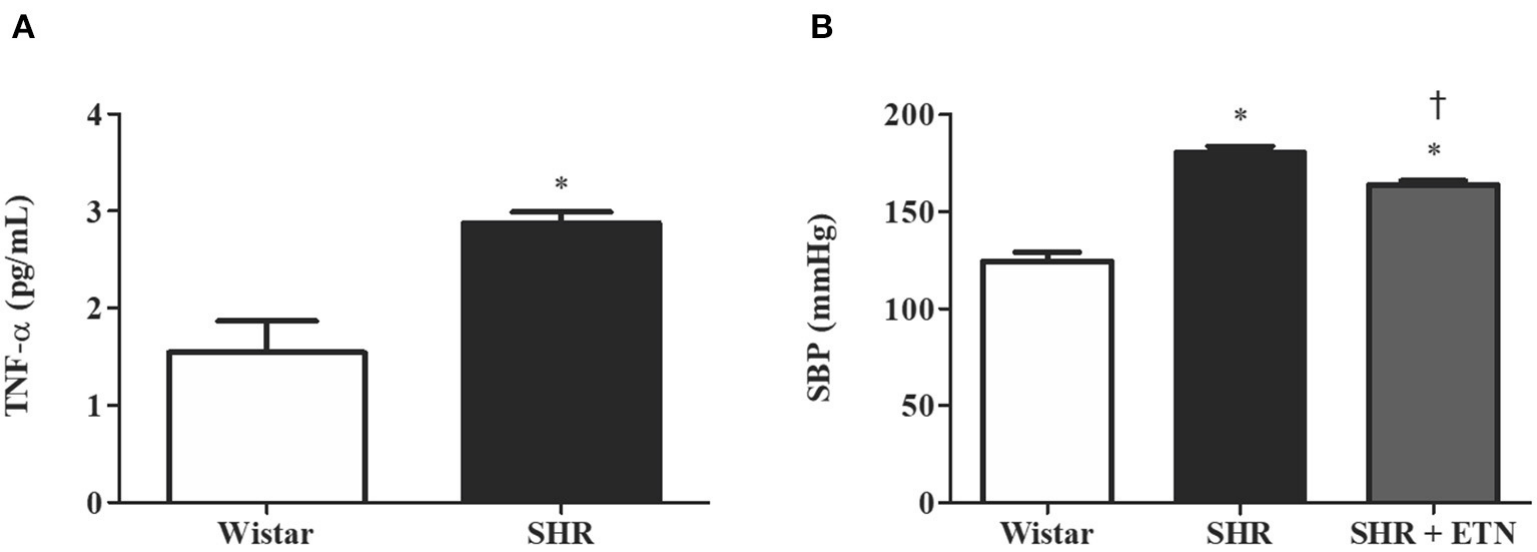
Placental tumor necrosis factor-alpha (TNF-α) levels and systolic blood pressure (SBP) are greater in spontaneously hypertensive rats (SHRs) compared to Wistar rats, and etanercept treatment reduces, without normalizing, SBP in treated SHRs. **(A)** Bar graph showing placental TNF-α levels (pg/ml) in SHR and Wistar; *n* = 6. **(B)** Bar graph showing SBP (mmHg) in SHR, Wistar, and SHR treated with etanercept; *n* = 5. Values are presented as means ± SEM, and data were analyzed by **(A)** Student's *t*-test or **(B)** one-way ANOVA, followed by Tukey posttest. *p < 0.05 vs. Wistar;^†^*p* < 0.05 vs. SHR.

Given the importance of the placenta in fetal nutrition, and considering that hypertension promotes low-grade inflammation, we analyzed placental TNF-α levels and confirmed the hypertensive pregnancy state by tail-cuff plethysmography. Furthermore, we treated a group of SHRs with etanercept, a TNF-α antagonist. Placental TNF-α levels were greater (3 ± 0.1 pg/ml vs. 1.5 ± 0.3 pg/ml; *p* = 0.003) and systolic blood pressure (SBP) was higher (181 ± 3 mmHg vs. 128 ± 5 mmHg; *p* < 0.0001) in SHRs compared to Wistar rats. Interestingly, when we treated SHRs with etanercept, the SBP was found to be reduced but not normalized [163 ± 2 mmHg; *p* = 0.003 (Figures 1A,B)].

### Aquaporin 3 Expression in Placental Tissue From Spontaneously Hypertensive Rats and Wistar Rats

**Figure 2:**
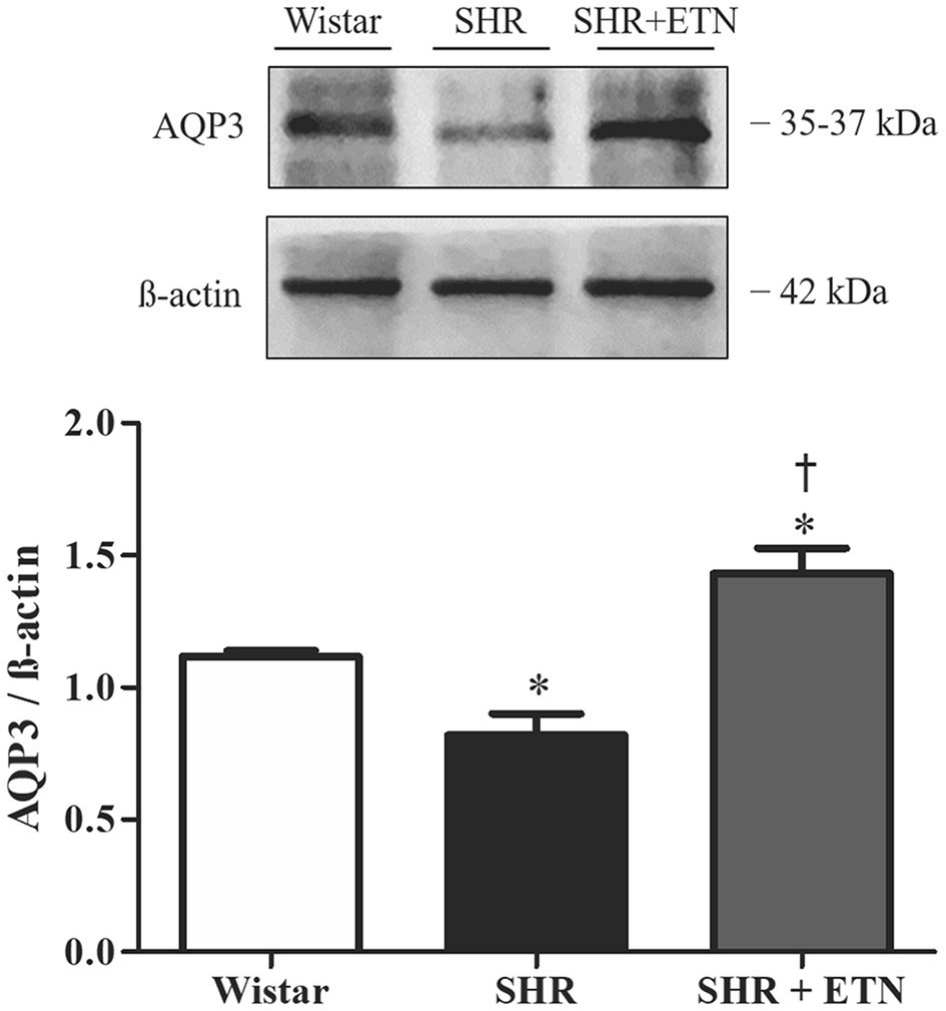
Placental aquaporin 3 (AQP3) expression is decreased in spontaneously hypertensive rats (SHRs) compared to Wistar rats, and etanercept treatment ameliorates SHR AQP3 expression. Upper representative picture of Western blot membrane with the respective AQP3 molecular weight. Bar graph showing the AQP3 expression in placentas from SHR, Wistar, and SHR treated with etanercept (ETN) after normalization to β-actin expression; *n* = 5. Values are presented as means ± SEM, and data were analyzed by one-way ANOVA, followed by Tukey posttest. *p < 0.05 vs. Wistar;^†^*p* < 0.05 vs. SHR.

To explore the role of TNF-α in AQP3 modulation in the placenta, we performed Western blot analysis of AQP3 in Wistar rats, SHRs, and SHRs treated with etanercept. Placentas from SHRs presented reduced AQP3 expression compared to Wistar rats. Interestingly, etanercept treatment elevated placental AQP3 expression (Figure 2). From here, we decided to investigate whether TNF-α levels were related to AQP3 in placental tissue using an *in vitro* model of human placenta explants.

### Aquaporin 3 Expression Upon Tumor Necrosis Factor-Alpha Incubation in Normal Human Placenta Explants

**Figure 3:**
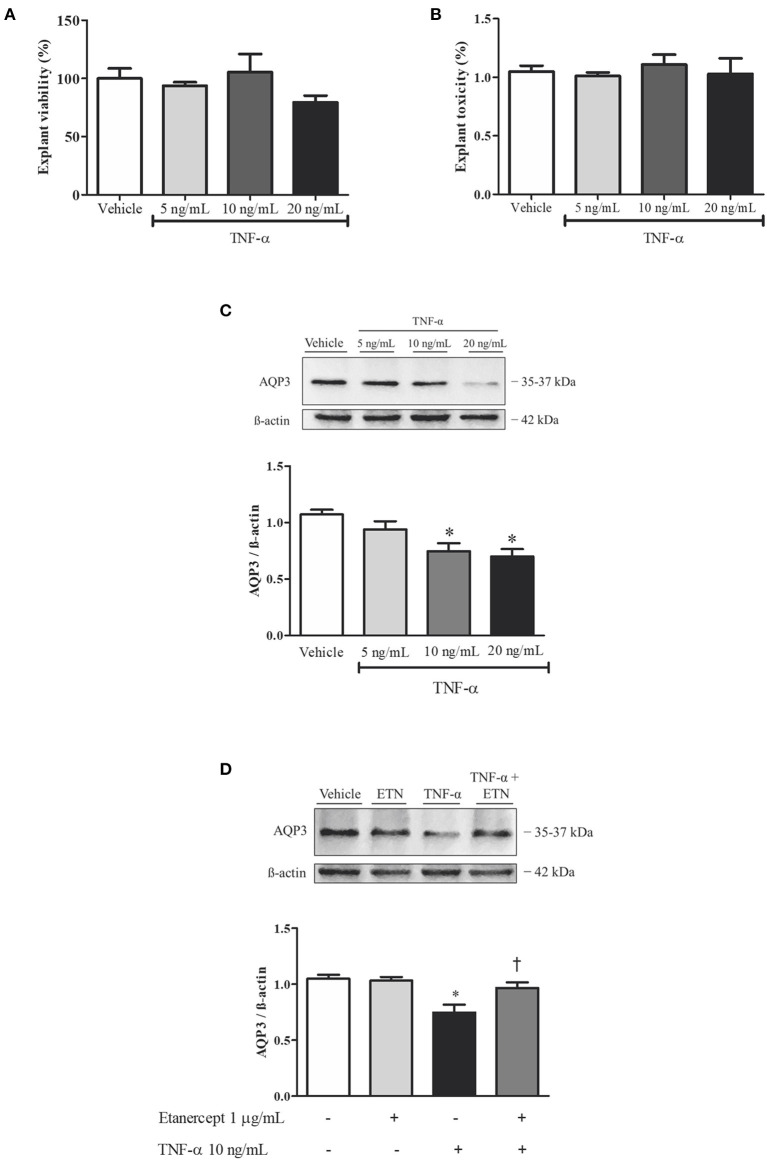
Tumor necrosis factor-alpha (TNF-α) incubation reduces aquaporin 3 (AQP3) expression in normal human placenta explants, and simultaneous incubation with TNF-α and etanercept (ETN) retrieves AQP3 expression. **(A,B)** Bar graphs showing explant viability and toxicity, respectively; *n* = 3. **(C)** Upper representative picture of Western blot membrane with the respective AQP3 molecular weight. Bar graphs showing the AQP3 expression upon incubation with vehicle (*n* = 7), 5 ng/ml TNF-α (*n* = 6), 10 ng/ml TNF-α (*n* = 6), and 20 ng/ml TNF-α (*n* = 7). **(D)** Bar graphs showing the AQP3 expression upon incubation with vehicle (*n* = 7), 1 μg/ml etanercept (*n* = 6), 10 ng/ml TNF-α (*n* = 6), and simultaneous TNF-α and etanercept (*n* = 6). Values are presented as means ± SEM, and data were analyzed by one-way ANOVA, followed by Tukey posttest. *p < 0.05 vs. vehicle;^†^*p* < 0.05 vs. 10 ng/ml TNF-α incubation.

Healthy human placental explants were incubated with different concentrations of TNF-α (5, 10, and 20 ng/ml) to evaluate AQP3 expression. Treatment with TNF-α did not affect explants' viability or toxicity (Figures 3A,B). TNF-α reduced the expression of AQP3 upon 10 and 20 ng/ml TNF-α incubation (Figure 3C). Additionally, simultaneous incubation with 10 ng/ml TNF-α and 1 μg/ml etanercept increased AQP3 expression (Figure 3D).

### Aquaporin 3 Expression and Cellular Migration in Extravillous Trophoblast Cells Incubated With Tumor Necrosis Factor-Alpha

**Figure 4:**
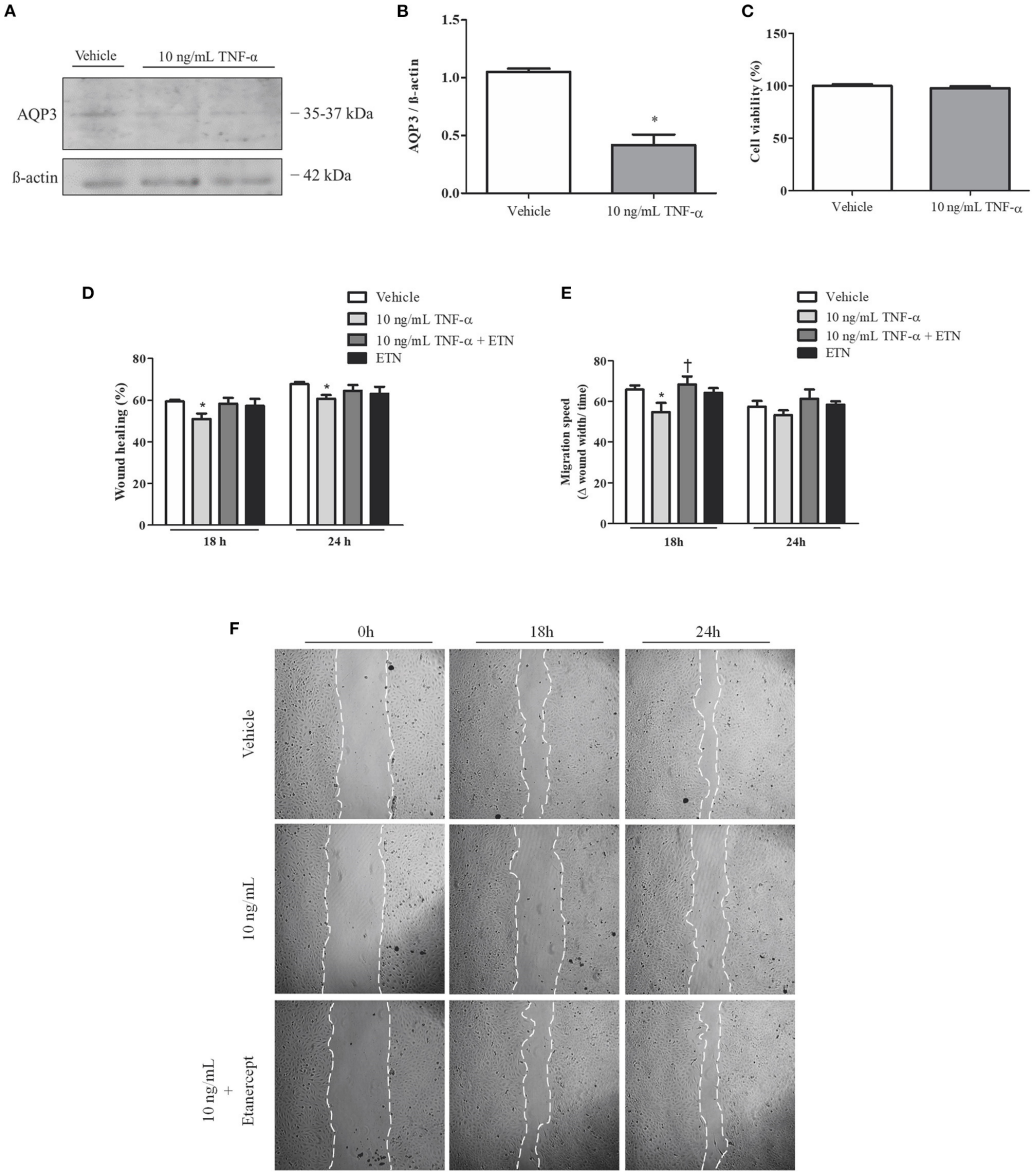
Tumor necrosis factor-alpha (TNF-α) reduces aquaporin 3 (AQP3) expression and impairs human trophoblastic cell migration. Concomitantly, simultaneous incubation with TNF-α and etanercept (ETN) ameliorates cell migration speed. **(A)** Representative picture of Western blot membrane with the respective AQP3 molecular weight. **(B)** Bar graph showing AQP3 expression in Swan 71 cells incubated with vehicle (*n* = 5) or 10 ng/ml TNF-α (*n* = 6). **(C)** Bar graph showing cellular viability (*n* = 5). **(D)** Bar graph showing the wound healing percentage in Swan 71 cells treated with vehicle (*n* = 6), 10 ng/ml TNF-α (*n* = 7), TNF-α + etanercept (*n* = 3), or 1 μg/ml etanercept (*n* = 2) after 18 and 24 h. **(E)** Bar graph showing the migration speed in Swan 71 cells treated with vehicle, 10 ng/ml TNF-α, TNF-α + etanercept, or 1 μg/ml etanercept after 18 and 24 h; *n* = 4. **(F)** Representative pictures of wound healing assay of Swan 71 cells incubated with or without 10 ng/ml TNF-α and 1 μg/ml etanercept after 0, 18, and 24 h. Values are presented as means ± SEM, and data were analyzed by two-way ANOVA, followed by Bonferroni posttest. *p < 0.05 vs. vehicle;^†^*p* < 0.05 vs. 18 h 10 ng/ml TNF-α incubation.

To investigate the role of TNF-α in EVT cell migration, Swan 71 cells were incubated with TNF-α and/or etanercept. AQP3 expression in Swan 71 cells was reduced after 10 ng/ml TNF-α incubation (Figures 4A,B). Additionally, *in vitro* experiments demonstrated that incubation with 10 ng/ml TNF-α impaired wound healing (Figures 4D–F). Concomitant incubation with TNF-α and 1 μg/ml etanercept improved the wound repair process. Moreover, incubation with TNF-α significantly decreased Swan 71 cell migration speed, and concomitant incubation with TNF-α and etanercept raised the migration rate (Figure 4E). Treatment with TNF-α did not affect cellular viability (Figure 4C).

## Discussion

The present study shows that hypertensive rats present decreased fetal weight and increased proportion of SGA fetuses concomitant with augmented placental levels of TNF-α and reduced placental AQP3 expression when compared to the normotensive group. Interestingly, etanercept treatment was able to both reduce BP levels and restore placental AQP3 expression in SHRs. The SHR animal model naturally shows an increase in BP after the 10th week of life (Breckenridge, 2013; Thoonen et al., 2015) mainly due to genetic predisposition to higher sodium. However, other mechanisms of BP increase have been proposed for SHRs, including a pro-inflammatory environment. During hypertension, a low-grade inflammatory condition is established with the secretion of inflammatory cytokines, including TNF-α (Mehaffey and Majid, 2017). This cytokine is majorly secreted by Th1 lymphocytes, and TNF-α is frequently involved in inflammatory and immunological processes occurring during arterial hypertension (Barbaro et al., 2015). Etanercept is a soluble recombinant TNF receptor that acts by inhibiting TNF-α action (Guillot et al., 2017). The reduced BP observed in SHRs treated with etanercept indicates that TNF-α plays a role in the maintenance of BP elevation, supporting the idea that TNF-α is part of the pathophysiology of high BP (Mehaffey and Majid, 2017; Lu and Crowley, 2018). Moreover, high levels of TNF-α are related to numerous obstetric complications, including recurrent abortions, failures in the implantation process, and PE (Saito et al., 2010).

AQPs are water channels playing a significant role in maintaining hydric homeostasis, allowing the rapid movement of water through the membranes (Roche and Tornroth-Horsefield, 2017). In this regard, AQPs play many roles in controlling cellular volume by being permeable to water but also, in some cases, to glycerol and urea (Ducza et al., 2017). Lately, AQPs have gained further interest since other features have been attributed to these water channels, including proliferation, apoptosis, and cell migration (Kitchen et al., 2015). In the placenta, AQPs are mainly expressed in the apical membrane of syncytiotrophoblast cells (Damiano et al., 2001). Although the exact function of AQPs in trophoblast cells remains unclear, some authors have recently shown that AQP3 blockade impairs EVT cell migration (Alejandra et al., 2018). Once AQP3 expression in SHR placenta is reduced, and considering that TNF-α inhibition ameliorated AQP3 placental expression, we investigated whether complications observed in hypertensive pregnancies may be due to AQP3. This hypothesis is further supported by the fact that AQP3 expression is reduced in placentas from human pregnancies complicated by PE (Szpilbarg and Damiano, 2017).

The inverse relation between TNF-α and AQP3 expression obtained in SHR placentas was further confirmed in human placentas. An *in vitro* study was performed by incubating normotensive human placental explants with TNF-α. Here, we aimed to test a concentration-dependent response using TNF-α at concentrations of 5, 10, or 20 ng/ml. Interestingly, TNF-α incubation negatively modulates AQP3 expression in placental explants, where both 10 and 20 ng/ml of TNF-α significantly reduced AQP3 expression. These data were confirmed when the explants were concomitantly incubated with TNF-α and 1 μg/ml etanercept, and the AQP3 expression was retrieved. Additionally, a previous study showed that TNF-α promoted a downregulation of AQP3 expression in human colonic adenocarcinoma (HT-29) cells through inhibition of constitutive transcriptional activity of the AQP3 promoter (Peplowski et al., 2017). Although 10 ng/ml TNF-α is a higher concentration than that observed in *in vivo* levels, it is in accordance with other studies using placental explants and is the most used concentration (Bauer et al., 2004; Leisser et al., 2006; Siwetz et al., 2016), providing the concentration range for TNF-α used in this study. Moreover, etanercept (1 μg/ml) incubation was used to inhibit the TNF-α actions before, as previously published by our group (Castro Parodi et al., 2011).

Human placental explants are constituted of placental villi, mainly composed of a specific trophoblastic cell type called cytotrophoblasts (CTBs). The arrangement of CTBs forms multinucleated syncytiotrophoblasts. During pregnancy progression, the CTB differentiates into the EVT, a cell type with migration properties that can invade the uterine wall. The reduced AQP3 expression observed in placental explants indicates that this event is happening before trophoblast differentiation into VT and EVT, probably being related to the most severe cases of PE, known as early-onset PE (Raymond and Peterson, 2011). This is the most critical subtype of PE, as it is associated with fetal growth restriction and is the major cause of PE-related morbidities and mortalities of mother and child (Huppertz, 2018). Moreover, the fact that hypertensive animals present both reduced fetal weight and AQP3 expression leads us to believe that deficient AQP3 is involved in fetal growth restriction. Interestingly, a previous study found that AQP3 null mice presented smaller fetuses, suggesting a role for AQP3 in fetal growth (Seo et al., 2018).

Although the complete mechanism eliciting PE remains unknown, its pathophysiology involves abnormal placentation and inadequate development of the uterine–placental vascular system (Williamson et al., 2017). Uteroplacental blood flow is allowed by a correct EVT invasion in uterine spiral arteries; however, prior to invasion into arteries, interstitial trophoblasts migrate deep into the uterus and reach the myometrium (Moser et al., 2018). This migration process is critical for EVT interaction with arteries and, consequently, for uteroplacental blood flow development (Pollheimer et al., 2018). In the present study, *in vitro*, TNF-α incubation impaired both EVT cell wound healing process and migration speed and reduced AQP3 expression in these cells. Concomitantly, simultaneous incubation with TNF-α and etanercept ameliorated EVT migration speed in these cells. These data indicate that TNF-α plays a deleterious role in migration by reducing AQP3 expression.

Besides TNF-α, the influence of other cytokines, especially the interleukins (ILs), on trophoblastic cell migration has been under study. IL-6, IL-8, IL-11, and IL-1β were found to stimulate *in vitro* trophoblast cell migration (Paiva et al., 2007; Hirota et al., 2009; Jovanovic and Vicovac, 2009; Jovanovic et al., 2010), while IL-27 incubation inhibited trophoblast cell migration and invasion (Ge et al., 2019). However, the relationship between these cytokines and AQP3 has not been established yet and must be addressed in future studies.

Evidence shows that AQPs may be involved in tumor cell migration (Cao et al., 2013; Ribatti et al., 2014). In cultured cancer cells, increased AQP3 expression increases cell proliferation, migration, and invasion, playing a pivotal and complex role in cancer progression (Marlar et al., 2017). Trophoblast cells of the human placenta use a very similar machinery for the cancer cells to grow, migrate, and invade but in a tightly regulated way (Soundararajan and Rao, 2004). In human placental trophoblastic cells, AQP3 inhibition or silenced AQP3 gene expression has reduced trophoblastic migration (Alejandra et al., 2018). Moreover, reduced AQP3 expression also reduces trophoblast endovascular differentiation and affects tubule formation (Reppetti et al., 2020). Our findings strongly support the hypothesis that TNF-α negatively impacts trophoblastic cell migration by reducing AQP3 expression. This observation is supported by the fact that TNF-α can impair *in vitro* trophoblast migration (Bauer et al., 2004).

In conclusion, the present study demonstrates, for the first time, the harmful modulation of AQP3 by augmented TNF-α levels in placental tissue, affecting trophoblast cell migration. This relationship is also observed in placental tissue from hypertensive pregnancy. Altogether, these observations contribute to a better understanding of placental impairment during hypertensive disorders of pregnancy.

## Data Availability Statement

The raw data supporting the conclusions of this article will be made available by the authors, without undue reservation.

## Ethics Statement

The studies involving human participants were reviewed and approved by Human Ethics Committee from the Faculty of Pharmacy and Biochemistry of the University of Buenos Aires. The patients/participants provided their written informed consent to participate in this study. The animal study was reviewed and approved by Committee of Ethics in Animal from the Federal University of Mato Grosso.

## Author Contributions

All authors listed have made a substantial, direct and intellectual contribution to the work, and approved it for publication.

## Conflict of Interest

The authors declare that the research was conducted in the absence of any commercial or financial relationships that could be construed as a potential conflict of interest.
